# Laparoscopy for evaluating mesenteric lymphangiomatosis: A case report

**DOI:** 10.3389/fonc.2022.933777

**Published:** 2022-10-20

**Authors:** Yefeng Yin, Rongdi Wang, Xishan Wang

**Affiliations:** ^1^ Department of Colorectal Surgery, National Cancer Center/National Clinical Research Center for Cancer/Cancer Hospital, Chinese Academy of Medical Sciences and Peking Union Medical College, Beijing, China; ^2^ Department of Anorectal Surgery, Dalian Municipal Central Hospital, Dalian, China

**Keywords:** lymphangiomatosis, exploratory, laparoscopy, colon, mesenteric

## Abstract

**Background:**

Lymphangiomatosis is an extremely rare disease with potential soft tissue, bone, and spleen involvement, which can be characterized by lymphangioma. Only a few cases of colon and mesenteric lymphangiomatosis have been reported. We report a case presenting with fatigue, periumbilical pain, and intermittent bloody stools. This patient underwent a series of examinations. Exploratory laparoscopy, in particular, yielded very valuable images and videos for this disease, which can provide evidence for the diagnosis of this disease.

**Case summary:**

The current patient had fatigue, periumbilical pain, and intermittent bloody stools. Colonoscopy indicated numerous variable-sized hyaline cysts in the colon. Submucosal puncture was performed during colonoscopy. The patient was readmitted to the hospital due to periumbilical pain. B-ultrasound and abdominal CT showed multiple hypoechoic nodules in the mesenteric area. Exploratory laparoscopy was performed, and histopathology revealed that D2-40 was positive. Based on auxiliary examination and laparoscopic biopsy, surgeons and pathologists reached the diagnosis of mesenteric lymphangiomatosis.

**Conclusion:**

Clinicians need to comprehensively improve their knowledge of lymphangiomatosis, and the combination of clinical symptoms, histological characteristics, and colonoscopy biopsy findings should be considered to improve lymphangiomatosis diagnosis, thereby reducing misdiagnosis.

**Core tip:**

Colon and mesenteric lymphangiomatosis is an extremely uncommon benign condition of unknown etiology and pathogenesis in adult patients. We report a case of mesenteric lymphangiomatosis in a 37-year-old woman who presented with fatigue, periumbilical pain, and intermittent bloody stools, as well as lesions in the kidney, spleen, and bones. This case provides new insights into the diagnosis and treatment of this disease.

## Introduction

Colon and mesenteric lymphangiomatosis is an extremely uncommon benign condition of unknown etiology and pathogenesis, which could lead to compression symptoms caused by mechanical pressure. Here, we report a case of abdominal lymphangiomatosis evaluated by laparoscopy, which has not been previously reported.

## Case presentation

**Figure 1:**
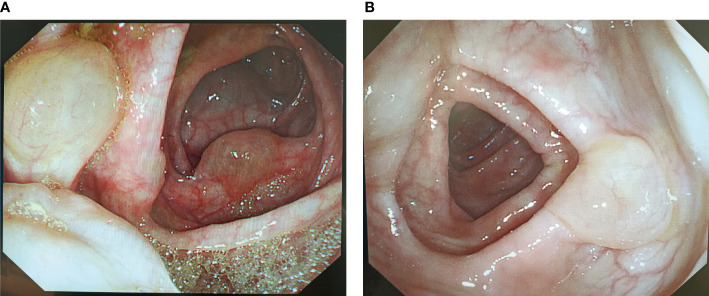
**(A)** Colonoscopy indicated numerous cysts in the ascending colon, transverse colon, and descending colon. **(B)** The patient treated with sirolimus within 3 months, and colonoscopy indicated that the number and size of cysts decreased.

**Figure 2:**
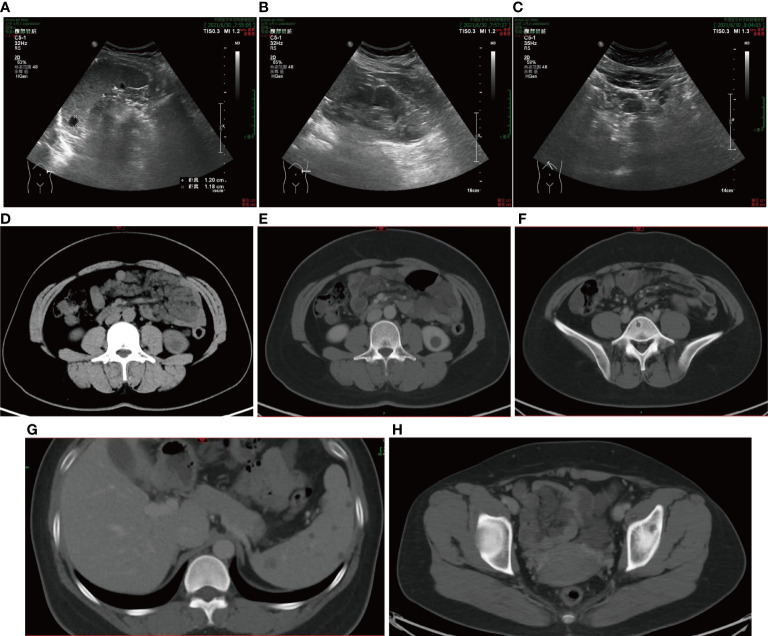
**(A–C)** Abdominal ultrasound observed multiple hypoechoic nodules in the spleen and the lower poles of the left kidney, and enlarged lymph nodes in the retroperitoneum. **(D)** Abdominal CT scan observed multiple diffuse low-density shadowing zones in the mesenteric area, which presented a doughnut shape. **(E–H)** Low-density shadowing zones can be observed in the left kidney, spleen, ilium, and the fifth lumbar vertebra in abdominal CT scan images.

**Figure 3:**
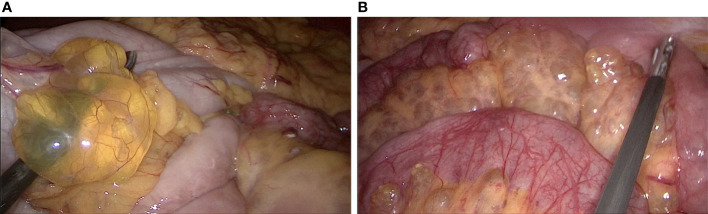
**(A, B)** Exploratory laparoscopy showed numerous hyalines, smooth-walled, variable-sized cystic microstructures in the root of the mesentery.

**Figure 4:**
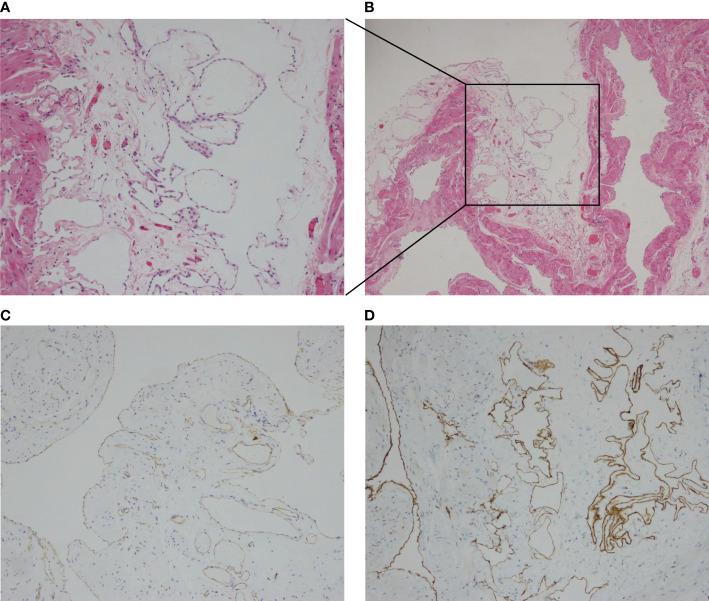
**(A, B)** Histological examination showed the positive D2-40 immunohistochemistry and confirmed mesenteric lymphangiomatosis. **(C, D)** Lymph vessels were identified imunohistochemically as D2-40-positive.

Colonoscopy indicated numerous variable-sized hyaline cysts, involving the ascending colon, transverse colon, and descending colon (**Figure 1A**), while no biopsy was carried out. Submucosal puncture was performed during colonoscopy. One year later, the patient returned with periumbilical pain. In abdominal ultrasound, multiple hypoechoic nodules with marked intranodular blood flow were observed in the spleen with a maximum size of 1.2 * 1.2 cm, with well-defined margins. In the inferior pole of the left kidney, a hypoechoic nodule was found, with an approximate diameter of 1.6 cm and no blood flow signal. Enlarged lymph nodes (1.9 * 1.1 cm^2^) were found in the retroperitoneum (**Figures 2A–C**). Abdominal CT revealed multiple diffuse low-density shadowing zones without enhancement distributed in the mesenteric area, which partly surrounded the small bowel and presented a doughnut shape. The boundaries were blurred (**Figure 2D**). Low-density shadowing zones were observed in the left kidney, spleen, ilium, and fifth lumbar vertebra on CT scan images (**Figures 2E–H**). Based on the images, lymphangiomatosis was highly suspected, which was further confirmed by laparoscopic biopsy (**Figure 3A**). Further exploratory laparoscopy showed numerous hyalines, smooth-walled, variable-sized cystic microstructures in the root of the mesentery, involving total mesenteric excision (**Figure 3B**). The liquid inclusions were clear and pale yellowish (**Video S1**). Histopathology revealed diffuse or multi-centric proliferation of thin-walled, dilated lymphatic vessels (**Figure 4A**). D2-40 (a monoclonal antibody), a highly sensitive and specific marker of lymphatic endothelium in the normal tissue and a subset of vascular lesions, confirmed mesenteric lymphangiomatosis by immunoreaction (**Figure 4B**). D2-40-positive lymphatic vessels were shown in mesentery (**Figures 4C, D**). The patient was treated with sirolimus (0.8 mg/m^2^, Bid) for 3 months, and follow-up colonoscopy was performed. Colonoscopy indicated decreased number and size of cysts compared with the condition observed 6 months before (**Figure 1B**).

## Discussion

In 2014, generalized lymphatic anomaly (GLA) was proposed by the International Society for the Study of Vascular Anomalies (ISSVA) as a new term for lymphangiomatosis (1).

Mesenteric lymphangiomatosis is a benign cystic tumor of lymphatic vessels that occurs rarely in adults, while its etiology and pathogenesis remain elusive (2). It has variable clinical presentations and can involve different sites, including the mesentery, bone, spleen, mediastinum, lungs, and soft tissues (3). The clinical course and compression symptoms are directly correlated to the affected sites, mechanical pressure, and extent of the disease. In most cases, these lesions are multicystic and characterized by hypodense regions on CT images, without increased vascular flow on color Doppler ultrasound. We analyzed 11 previous cases of lymphangiomatosis involving the gastrointestinal tract and summarized them in **Table 1**. We listed the symptom and examination procedure of these cases so that colleagues can learn more about the disease. Reviews of the literature confirm that lymphangiomatosis progresses slowly and can be well controlled with appropriate treatment.

**Table 1:** Previous lymphangiomatosis-related disease cases.

Case number	Gender	Age	Lesion location		Clinical features	Diagnostic workup	Treatment	Follow-up and outcome	Reference
1	Male	59	Esophagus		Choking on swallowing	Endoscopy: multiple submucosal masses, slightly translucent and whitish, covered with smooth and normal-looking mucosa; Chest CT: diffuse low-density lesion; Endoscopic ultrasound: multiple mixed solid and anechoic cystic lesions above the seemingly intact muscularis propria; Histopathologic: lymphangioma	Observation	Progression-free	Cao et al. (4)
2	Male	79	Sigmoid colon		Intermittent bloody stools, abdominal discomforts	Fecal occult blood test: positive; Colonoscopy: multiple cystic masses with a translucent and smooth surface, ranging from 4 to 8 mm in diameter; EUS: echo-free cystic masses	Laparoscopy-assisted partial sigmoid colon resection	Progression-free	Lu et al. (5)
3	Male	52	Ascending colon		Intermittent bloody stools, abdominal discomforts	Endoscopy: large, balloon-like submucosal structures; Histology: nonspecific mild chronic inflammation	Observation	Disease progression	Mujagic et al. (6)
4	Male	41	Ileum		Acute diffuse abdominal pain, nausea, vomiting and inability to pass gas or stool	Abdominal CT scan: bowel distension and multiple gas-fluid levels; Diagnostic laparoscopy:ileal perforation; Histology: diffuse lymphangiomatosis involving the sub-mucosa and, in some parts, the full-thickness muscular wall	Ileal resection	Not mentioned	Giuliania et al. (7)
5	Male	71	Jejunum		The patient underwent rectal resection and ileostomy. Follow-up examination revealed nodular jejunal and adjacent mesenteric masses	Abdominal CT scan: soft-tissue density of the nodular mass and hazy attenuations in the jejunal mesentery; Contrast-enhanced CT: nodular jejunal and adjacent mesenteric masses in contrast to the barium-filled jejunum; PET/CT: no remarkable FDG uptake was seen; Histology: cavernous lymphangioma involving the jejunum and mesentery.	Jejunectomy	Progression-free	Hwang and Park (8)
6	Male	31	Jejunum, Cecum		Recurrent melena for the last 8 years and iron deficiency anemia	Colonoscopy: protruding submucosal lesions of approximately 20–30 mm in diameter at the cecum; Histology: mild mononuclear infiltrate in lamina with focal prominence of goblet cells; Contrast-enhanced CT: small cystic lesion in ascending colon; Laparotomy: a leash of blood vessels and multiple fleshy sessile pedunculated lesions overlying serosal aspect of small bowel and small bowel mesentery starting from mid-jejunum to the ileo-cecal junction	Limited ileocecal resection	Progression-free	Rai et al. (9)
7	Male	58		Esophagus	Dysphagia of 7 months’ duration	Esophagogastroscopy: a huge lesion grew along the esophagus; the mucosa was normal; Esophageal ultrasonography: the tumor originated from the submucosal layer and the muscularis propria was found to be intact; Contrast-enhanced CT: a mass localized inside the esophageal lumen and a lesion with low density was outside; Histology (endoscopic biopsy specimen): mild to moderate dysplasia of the lesion mucosa; Histology (resection specimen): lymphangioma	Open thoracotomy and enucleation	Progression-free	Liang et al. (10)
8	Male	32		Small bowel mesentery	Mild dull diffuse abdominal pain and two episodes of melena	Abdominal ultrasound: multiple anechoic cystic lesions within the abdominal cavity; Abdominal CT: numerous confluent cystic lesions of variable size; Cytology: few mature lymphocytes and was negative for chyle. Flow cytometry showed few B and T lymphocytes; Endoscopy: no significant abnormality.	1 g of oral paracetamol three times per day for 1 week	Progression-free	Alhasan and Daqqaq (11)
9	Female	35		Mediastinum, Abdomen	Abdominal pain, lymphoedema of the legs, ascites, and diarrhea	Thoracoabdominal region CT: a multilobar cystic mass in the mediastinum extending in the retroperitoneum and intraperitoneum; Body MRI: multiple hyperintense cystic lesions in mediastinum and retroperitoneum on T2-weighted images; Endoscopy: multiple white spots in the duodenum and jejunum. Histology:no evidence of malignancy	No effect: Pan abdominal radiotherapy, sandostatin, doxycycline, beta-blockade and thalidomide; Effect: sirolimus	Progression-free	Van Meerhaeghe et al. (12)
10	Female	Newborn		Right lower abdominal subcutis	None	Abdominal ultrasound: hypo-anechoic cysts in the subcutaneous tissues, extending from the umbilical to the right inguinal area, reaching the medial surface of the thigh; Abdominal MRI: several lacunas with cystic appearance; the enhancement was absent. Multicystic lymphangioma was diagnosed	“Wait and see” strategy, follow-up 3 years.	Progression-free	Amodeo et al. (13)
11	Female	11		Mesentery	Pain in the right lower quadrant of the abdomen for 2 months	Abdominal CT: infiltrative cystic mass in the mesentery; Exploratory laparotomy: a broad-based multiple cystic lymphangioma within the mesentery ranging from the Treitz ligament to the transverse colon.	The lymphangiomawas completely excised saving mesenteric vessels by skeletonization. The mesenteric defect was repaired with a Permacol.	Progression-free	Kim et al. (14)

A case report of mesenteric lymphangiomatosis indicated that surgical resection of the mesentery could be an option in case of mechanical obstruction (15). However, the defect caused by the operation may lead to internal herniation, eventually resulting in bowel obstruction and intestinal volvulus. Repairing the defect with Permacol was performed with no complications after 4 years (14). Biomaterial implant may be an option for closing the mesenteric defect caused by surgery. Lymphorrhea after open or laparoscopic biopsy is one of the possible complications. Minor lymphatic leakage can be resolved by conservative management. Massive lymphatic leaks can be treated by embolization, but surgery may be necessary in some cases (16). Regarding the pharmacotherapy of lymphangiomatosis, sirolimus is an inhibitor of mammalian target of rapamycin (mTOR). Elisa Boscolo reached the conclusion that sirolimus suppresses the growth of lymphatic endothelial cells by inhibiting VEGF-A- and VEGF-C-driven proliferation and migration, thus impeding lymphangiogenesis (17). It is very effective in the treatment of multiple complicated vascular anomalies and has potential antitumor effects (18). Follow-up of the present case observed a significant decrease in number and size of cysts by colonoscopy.

Exploratory laparoscopy has not been utilized to evaluate mesenteric lymphangiomatosis so far, although a case report suggested that surgical resection of the mesentery could be an option in case of mechanical obstruction (14). The present case revealed the multi-systemic imaging manifestations of a rare systemic disease and highlighted the importance of exploratory laparoscopy in the diagnosis of mesenteric lymphangiomatosis.

## Data availability statement

The datasets presented in this study can be found in online repositories. The names of the repository/repositories and accession number(s) can be found in the article/**Supplementary Material**.

## Author contributions

XW and YY contributed to the study concept and design. YY generated the literature strategy and filtered through the identified studies. YY evaluated study quality and wrote the manuscript. RW provided critical feedback on the manuscript. All authors contributed to the article and approved the submitted version.

## Funding

National Natural Science Foundation of China, Grant Number: 82072732, 81572930; The National Key Research and Development Program of China, Grant Number: 2016YFC0905303; Beijing Science and Technology Plan, Grant Number: D171100002617004.

## Acknowledgments

We thank Dr. Hulin Ma for data acquisition and clinical assessment and thank pathologist Quan Zhou for her contribution in the diagnosis of mesenteric lymphangiomatosis.

## Conflict of interest

The authors declare that the research was conducted in the absence of any commercial or financial relationships that could be construed as a potential conflict of interest.

## Publisher’s note

All claims expressed in this article are solely those of the authors and do not necessarily represent those of their affiliated organizations, or those of the publisher, the editors and the reviewers. Any product that may be evaluated in this article, or claim that may be made by its manufacturer, is not guaranteed or endorsed by the publisher.

## Supplementary material

Supplementary Video 1 Laparoscopic presentation of lymphangiomatosis.
